# The Plasma Membrane H^+^ ATPase CsPMA2 Regulates Lipid Droplet Formation, Appressorial Development and Virulence in *Colletotrichum siamense*

**DOI:** 10.3390/ijms242417337

**Published:** 2023-12-11

**Authors:** Yu Liu, Yitao Xi, Yanyu Lv, Jingting Yan, Miao Song, Hong Yang, Yu Zhang, Weiguo Miao, Chunhua Lin

**Affiliations:** 1 Sanya Institute of Breeding and Multiplication, School of Tropical Agriculture and Forestry, Hainan University, Haikou 570228, China; liuyuya0312@163.com (Y.L.); lvyanyun0323@163.com (Y.L.); yanjingting958@163.com (J.Y.); songmiao0614@163.com (M.S.); yanghong@catas.cn (H.Y.); yuzhang_rain@163.com (Y.Z.); miao@hainanu.edu.cn (W.M.); 2 Rubber Research Institute, Chinese Academy of Tropical Agricultural Science, Haikou 571101, China; xi10007@126.com

**Keywords:** *Colletotrichum siamense*, plasma membrane ATPases, CsPMA2, lipid droplets, appressorium development, pathogenicity

## Abstract

Plasma membrane H^+^-ATPases (PMAs) play an important role in the pathogenicity of pathogenic fungi. Lipid droplets are important storage sites for neutral lipids in fungal conidia and hyphae and can be used by plant pathogenic fungi for infection. However, the relationship between plasma membrane H^+^-ATPase, lipid droplets and virulence remains unclear. Here, we characterized a plasma membrane H^+^-ATPase, CsPMA2, that plays a key role in lipid droplet formation, appresorial development and virulence in *C. siamense*. Deletion of *CsPMA2* impaired *C. siamense* conidial size, conidial germination, appressorial development and virulence but did not affect hyphal growth. Δ*CsPMA2* increased the sensitivity of *C. siamense* to phytic acid and oxalic acid. CsPMA2 was localized to lipids on the plasma membrane and intracellular membrane. Deletion of *CsPMA2* significantly inhibited the accumulation of lipid droplets and significantly affected the contents of some species of lipids, including 12 species with decreased lipid contents and 3 species with increased lipid contents. Furthermore, low pH can inhibit *CsPMA2* expression and lipid droplet accumulation. Overall, our data revealed that the plasma membrane H^+^-ATPase CsPMA2 is involved in the regulation of lipid droplet formation and affects appressorial development and virulence in *C. siamense*.

## 1. Introduction

Plasma membrane P-type ATPase is a type of transport protein located on the plasma membrane that can drive material transport through energy-consuming ATP hydrolysis reactions [1,2]. These proteins play important roles in physiological processes such as intracellular and extracellular material transport, cell volume regulation, and ion balance [3,4]. According to the properties of its transport substances, the plasma membrane transport P-type ATPase can be divided into multiple subtypes, including sodium potassium pump (Na^+^/K^+^-ATPase), calcium pump (Ca^2+^-ATPase), proton pump (H^+^-ATPase), and heavy metal pump [5,6]. Among these subtypes, plasma membrane H^+^-ATPase is a type of hydrogen proton pump driven by ATP, which is responsible for pumping hydrogen protons from the cytoplasm to the extracellular space to maintain intracellular pH and ion homeostasis, form transmembrane electrochemical potential, and promote nutrient transportation and the expulsion of secondary metabolites [7,8,9]. Most fungi have two plasma membrane H^+^-ATPases, PMA1 and PMA2 [10,11,12,13]. The structure and function of plasma membrane H^+^-ATPases, especially PMA1, have been extensively studied in some fungi, such as *Fusarium graminearum*, *Valsa mali,* and *Ustilago maydis* [14,15]. Most studies have shown that PMA1 is localized to lipid microdomains of the plasma membrane and plays a central role in the regulation of the pH homeostasis system, and it is responsible for cell growth and virulence in pathogenic fungi [16,17], such as *PMA1* in *Neurospora crassa* [18], *PMA1* in *F. graminearum* [19], *PMA2* in *Magnaporthe grisea* [20], *PMA1* in *Leptosphaeria maculans* [21], and *PMA1* in *U. maydis* [17]. Unlike PMA1, it is generally believed that PMA2 has little effect on the growth and pathogenicity of fungi due to its extremely low expression in cells [22]. For example, *FgPMA2* in *F. graminarium* and *LmPMA2* in *L. maculans* have shown that the *PMA2* homolog does not affect vegetative growth, sexual and asexual development, or virulence [19,21]. However, it has been reported that *ChPMA2* in *Colletotrichum higginsiamum* is needed for appressorial formation and infection [23]. However, the regulatory mechanism of *PMA2* on virulence is still unclear.

Lipid droplets (LDs) are ubiquitous organelles that store neutral lipids for energy of membrane synthesis and act as hubs for metabolic processes [24,25]. The aggregation of lipid droplets is closely related to pH. Relevant studies have shown that lipid droplet aggregation is relatively stable at pH 6–7, which makes it extremely unstable under acidic conditions due to the strong electrostatic repulsion between lipid droplets in this pH range [26]. Studies in *Saccharomyces cerevisiae* also found that changes in the pH of the external environment would affect lipid droplet homeostasis, further affect lipid droplet lipolysis, lead to changes in the glycerol and fatty acid content of the bacteria, and then affect a range of intracellular lipid metabolisms. In addition, there is also a physiological relationship between pH and phospholipid metabolism, and the rapid decrease in pH will damage the electrostatic interaction between PA and Opi1, and affect the transcription of a number of phospholipid metabolism genes [27]. It is worth noting that the process of lipid droplet formation and degradation is also closely related to the homeostasis of intracellular lipids. In human cells, when the biosynthesis and degradation of lipid droplets are imbalanced, intracellular lipids begin to accumulate, promoting the activation of the pathogenic mechanism leading to steatosis, hepatocyte inflammation and fibrosis. Lipid metabolites generated by the hydrolysis of TG and other esterified neutral substances from lipid droplets and intermediates of TG synthesis will affect cell balance and induce organelle dysfunction, cell injury, cell dysfunction and death [28].

Previous studies have shown that LDs are important storages and can be used by pathogenic fungi for infection [29,30,31]. Most fungi infect their host through a specialized structure called the appressorium [32]. During appressorium maturation, LDs are transported into appressorial vacuoles where they are degraded by triacylglycerol lipase during turgor generation [30,33]. Many lipid droplet-associated proteins have been identified to be involved in LD physical stability and lipid digestibility. These proteins often play roles in appressorial development and affect virulence in pathogenic fungi. For example, MPL1 in *Metarhizium anisopliae* and Cap20 in *Colletotrichum gloeosporioides* encode lipid droplet coat proteins and play an important role in lipid metabolism, appressorial turgor pressure and virulence [24,34,35]. Nem1 in *Magnaporthe oryzae* is also a lipid droplet-associated protein that plays a key role in LD biogenesis and regulates appressorial function during infection [31]. Studies have shown that there may be an interplay between lipids and PMA1 in fungal cells. PMA1 has been reported to be localized in the lipid microdomains of the plasma membrane, and studies in *Saccharomyces cerevisiae* have shown that the efficient function of PMA1 is greatly dependent on sphingolipids [27,36]. Abnormally high ergosterol accumulation at the plasma membrane disrupts the structures of lipid rafts, resulting in the mislocalization of PMA1 in cells [15,27,37]. However, the relationship between plasma membrane H^+^-ATPase and LDs is not fully understood.

*Colletotrichum* spp. are one of the dominant causal agents of plant diseases that can cause extensive yield losses of major agricultural crops worldwide [38,39]. *Colletotrichum* is a fungus that is able to penetrate the cuticles of host plants by generating appressoria [40,41,42,43], which develop enormous turgor pressure to allow for cuticle penetration [44]. In this study, we identified a plasma membrane H^+^-ATPase, CsPMA2, in *Colletotrichum siamense* (one of the dominant pathogenic species of many tropical or subtropical crops) and found that it was localized to lipids and involved in LD formation, appressorial turgor pressure formation and virulence. Our results not only enrich the understanding of the function of the plasma membrane H^+^-ATPase PMA2 but also reveal a novel regulatory mechanism of LDs during appressorial formation in *C. siamense*.

## 2. Results

### 2.1. Identification and Deletion of the CsPMA2 Gene in C. siamense

**Figure 1:**
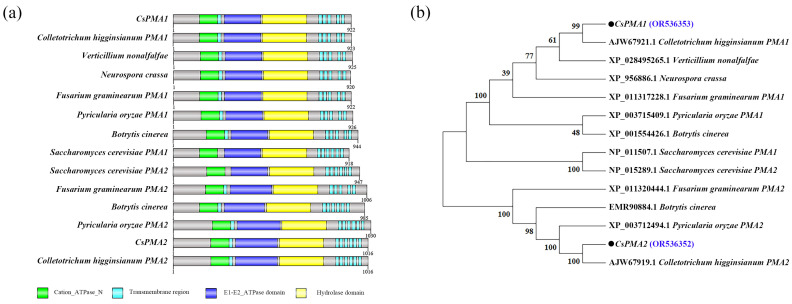
Protein domains and phylogenetic analysis of CsPMA2. (**a**) Protein domains of CsPMA1, CsPMA2 and their homologs. (**b**) Phylogenetic tree of CsPMA2 and its homologs. The phylogenetic tree was constructed with MEGA 11.0.10 software using the maximum likelihood method. The bootstrap values from 1000 replications are given at the branches of the tree. The two plasma membrane transport proteins identified in *C. Siemens* were marked in blue font.

We found two *C. siamense* plasma membrane H^+^ATPase genes, *CsPMA1* and *CsPMA2*, via a local BLAST search of the *C. siamense* HN08 genome database and transcription database in our laboratory. Sequence analysis showed that the *CsPMA2* gene has a DNA size of 3354 bp and a cDNA size of 3051 bp, encoding a 1016 amino acid peptide with a Cation_ATPase_N domain, six transmembrane domains, an E1-E2_ATPase domain, and a hydrolase domain (Figure 1a). Phylogenetic clustering showed that the CsPMA2 amino acid sequence shared 45.74% identity with *S. cerevisiae* PMA2 and high identity with other fungal PMA2 proteins or homologs (Figure 1b). This verified that the sequence obtained was a plasma membrane H+-ATPase PMA2 homolog in *C. siamense*. We named it *CsPMA2* and deposited the sequence into GenBank (Accession No. OR536352).

To investigate the function of *CsPMA2*, we deleted *CsPMA2* in *C. siamense* HN08 using a homologous recombination strategy (Figure S1a). Two independent deletion mutants, Δ*CsPMA2*-5 and Δ*CsPMA2*-19, were obtained and confirmed by PCR amplification (Figure S1b) and sequencing. The internal sequence of the *CsPMA2* gene could not be amplified by the primers CsPMA2-F/CsPMA2-R, and the products were amplified by the primers CsPMA2-OU-F/ILV1-R and ILV1-F/CsPMA2-OD-R in the ∆*CsPMA2* mutants but not in the wild type. The PCR products amplified from mutant Δ*CsPMA2*-5 and Δ*CsPMA2*-19 and wild-type HN08 using primers CsPMA2-OU-F/CsPMA2-OD-R were sequenced, yielding a 4801 bp fragment from Δ*CsPMA2*-5 and Δ*CsPMA2*-19 and a 5338 bp fragment from HN08. This result indicated that the 3354 bp open reading frame of the *CsPMA2* gene was replaced by the 2817 bp fragment of the *ILV1* gene, as in the design shown in Figure S1a. We also obtained complementary strains by introducing the vector pBAR-CsPMA2-GFP, which contains the coding region of *CsPMA2* driven by the promoter GpdA. The complementary strains were verified by PCR amplification (Figure S1c), one of them was chosen for further analysis, and its phenotypes were all similar to those of HN08.

### 2.2. CsPMA2 Deletion Mutants Showed Small Conidia, Decreased Conidial Germination and Appressorium Formation Rates, and Retarded Appressoria Development in C. siamense

**Figure 2:**
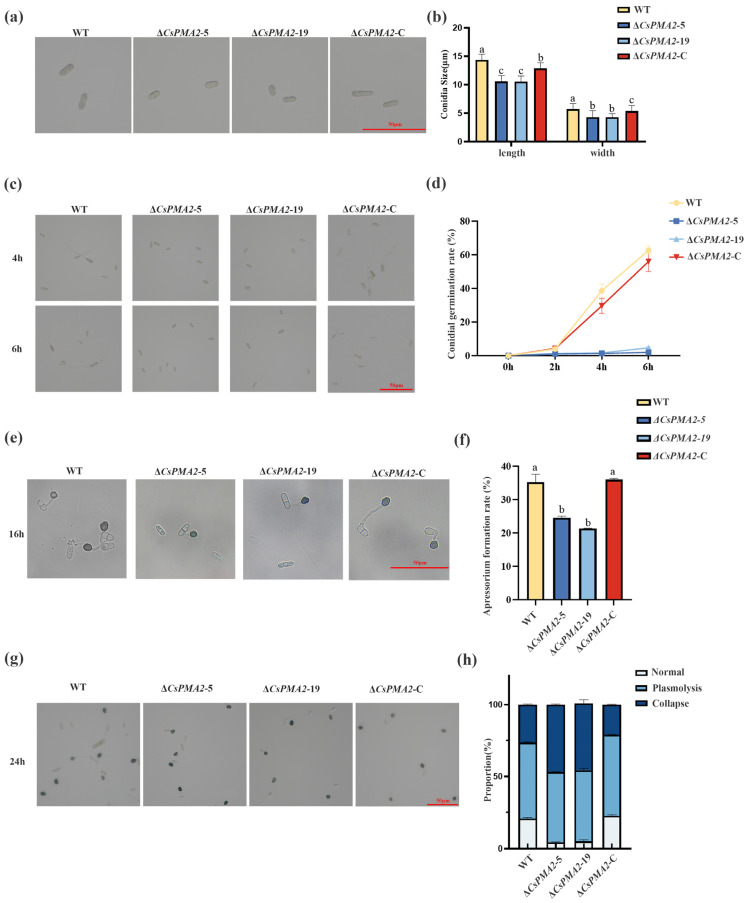
Comparison of conidial size, conidial germination rate, appressorial formation rate and appressorial turgor of wild-type HN08, ∆*CsPMA2*-5, ∆*CsPMA2*-19, and Δ*CsPMA2*-C. (**a**,**b**) Morphology and conidial size of the tested strains; (**c**,**d**) Morphology and conidial germination rates of the tested strains; (**e**,**f**) Morphology and appressorial formation rates of the tested strains. (**g**,**h**) Plasmolysis rates and collapse rates of appressoria of the tested strains treated with 0.8 g/mL PEG8000. Different letters indicate an extremely significant difference (*p* < 0.01) (one-way ANOVA and Duncan’s test), and error bars represent the standard deviations.

**Figure 3:**
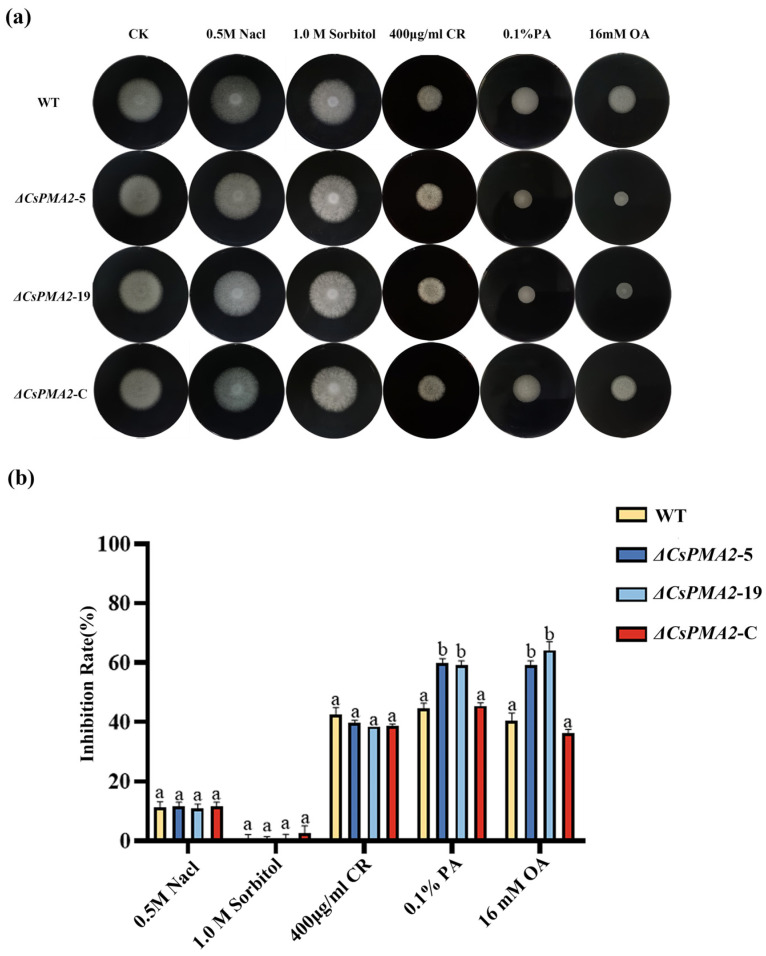
Comparison of responses to various stresses among wild-type HN08, ∆*CsPMA2*-5, ∆*CsPMA2*-19, and Δ*CsPMA2*-C. (**a**) Mycelial growth of the tested strains on CM supplemented with 0.5 NaCl, 1 M sorbitol, 400 µg/mL Congo Red (CR), 0.1% phytic acid (PA) and 16 mM oxalic acid (OA) for 3 days; (**b**) Growth inhibition rates of the tested strains under various stresses. The growth inhibition rate was calculated using the following formula: Inhibition rate (%) = [(control diameter − treated diameter)/(control diameter × 100%)]. Each treatment had three replicates. Error bars represent the standard deviations. Different letters indicate an extremely significant difference (*p* < 0.01) (one-way ANOVA and Duncan’s test).

To characterize the biological function of the *CsPMA2* gene in *C. siamense*, the colony morphology, mycelial growth, conidial size, conidial germination, and appressorial development of Δ*CsPMA2-*5 and Δ*CsPMA2-*19*,* Δ*CsPMA2*-C, and the wild-type (HN08) strains were compared (Figure 2 and Figure 3a). No significant difference in the morphology of colonies and mycelial growth rate of the four tested strains was observed on CM plates (Figure 3a). However, conidia sizes, conidia germination rate and appressorial formation rate and appressorial turgor pressure generation of gene deletion mutants were retarded significantly. The conidia sizes of Δ*CsPMA2-*5 and Δ*CsPMA2*-19 were (10.57 ± 1.26) × (4.98 ± 0.73) µm and (10.56 ± 0.97) × (5.01 ± 0.84) µm, and those of the wild type and Δ*CsPMA2*-C were (14.34 ± 3.61) × (5.70 ± 0.88) µm and (12.81 ± 1.65) × (5.32 ± 0.69) µm, respectively (Figure 2a,b). The conidial germination rates of Δ*CsPMA2*-5 and Δ*CsPMA2*-19 were 6.00% and 7.00% at 6 h, respectively, while those of the wild type and Δ*CsPMA2*-C were 50.00% and 50.33%, respectively (Figure 2c,d). The appressorial formation rates of Δ*CsPMA2*-5 and Δ*CsPMA2-*19 were 24.62% and 21.34% at 16 h, respectively, while those of the wild type and Δ*CsPMA2*-C were 35.33% and 36.05%, respectively (Figure 2e,f). Furthermore, the appressoria were incubated with 0.8 g/mL PEG8000 for 10 min, and the appressorial plasmolysis and cytorrhysis rates were analyzed. The results showed that the appressorial plasmolysis rates were 48.76%, 49.37%, 56.21% and 52.95% for the Δ*CsPMA2-*5 and Δ*CsPMA2-*19*,* Δ*CsPMA2-*C, and HN08 strains, respectively (Figure 2g,h). The appressorial cytorrhysis rates were 46.91%, 45.46%, 21.14% and 26.41% for the Δ*CsPMA2*-5, Δ*CsPMA2*-19*,* Δ*CsPMA2*-C, and HN08 strains, respectively (Figure 2g,h). This finding indicated that the appressorial turgor pressure of the mutants was lower than that of the wild-type strains, and the cell wall porosity of the mutant appressoria was greater than that of the wild-type strains. Overall, these data indicated that the loss of the *CsPMA2* gene affected the conidia size and decreased the conidia germination rate and appressorial development in *C. siamense*.

### 2.3. CsPMA2 Gene Is Involved in the Response to Acid Stress

Considering that plasma membrane H^+^-ATPase is involved in maintaining intracellular pH and ion homeostasis, we investigated whether *CsPMA2* is involved in acid stress sensitivity. The sensitivities of Δ*CsPMA2*-5 and Δ*CsPMA2-*19, Δ*CsPMA2*-C, and the wild-type (HN08) strains on CM media with different agents were measured (Figure 3). No significant difference in growth inhibition were observed between the four tested strains when they were exposed to 0.5 M NaCl, 1.0 M sorbitol, 400 μg/mL Congo Red, which indicated that the *CsPMA2* gene did not affect the osmotic stress response of *C. siamense*. However, Δ*CsPMA2*-5 and Δ*CsPMA2*-19 exhibit more sensitive characteristics than the wild-type strain HN08 under 0.1% PA and 16 mM OA treatments. The growth inhibition of Δ*CsPMA2*-5, Δ*CsPMA2*-19, Δ*CsPMA2*-C and HN08 was 59.85%, 59.32%, 45.47% and 44.57% under 0.1% phytic acid stress, respectively. The growth inhibition of Δ*CsPMA2*-5, Δ*CsPMA2-*19, Δ*CsPMA2-*C and HN08 was 59.17%, 64.17%, 36.25% and 40.42% under 16 mM oxalic acid stress, respectively. These data showed that the *CsPMA2* gene is involved in the response to acid stress, which is consistent with the finding that the plasma membrane H^+^ P-type ATPase plays a role in intracellular pH homeostasis.

### 2.4. CsPMA2 Deletion Attenuates the Virulence of C. siamense

**Figure 4:**
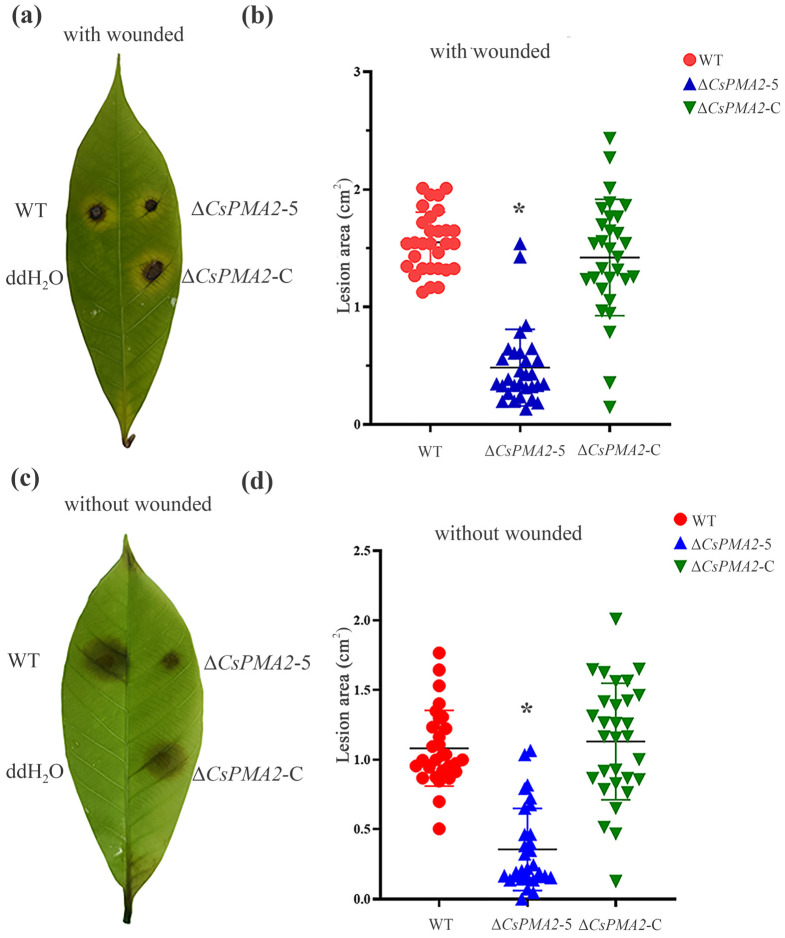
Virulence assays on rubber tree leaves. (**a**,**c**). Symptoms of the virulence assays of the tested strains. The rubber tree leaves were inoculated with 20 µL of conidial suspension (1 × 10^5^ spores/mL) of tested strains through unwounded and wounded methods. (**b**,**d**) The dot plot analysis of the lesion areas is shown at 5 days after inoculation. Thirty leaves were inoculated per treatment (* *p* < 0.1, according to one-way ANOVA and Duncan’s test; the error bar shows the standard deviation value).

The pathogenicity of Δ*CsPMA2*-5, Δ*CsPMA2*-C and HN08 was comparable on healthy light green rubber tree leaves that were wounded or nonwounded. The lesion area was measured 5 days after inoculation (Figure 4). On the wounded leaves, all four tested strains caused disease symptoms, and the diseased area caused by Δ*CsPMA2*-5 (mean 0.48 ± 0.36 cm^2^) was smaller than those caused by the HN08 strain (mean 1.55 ± 0.45 cm^2^) and ∆*CsPMA2*-C strain (mean 1.42 ± 0.78 cm^2^) (Figure 4a,b). On the nonwounded leaves, the diseased area caused by ∆ *CsPMA2*-5 (mean 0.35 ± 0.71 cm^2^) was also significantly smaller than that of the HN08 strain (mean 1.00 ± 0.77 cm^2^) and ∆*csPMA2*-C strain (mean 1.13 ± 0.87 cm^2^) (Figure 4c,d). The results indicated that the lack of the *CsPMA2* gene affects the virulence of *C. siamense.*

### 2.5. CsPMA2 Is Localized to Lipids on the Plasma Membrane and Intracellular

**Figure 5:**
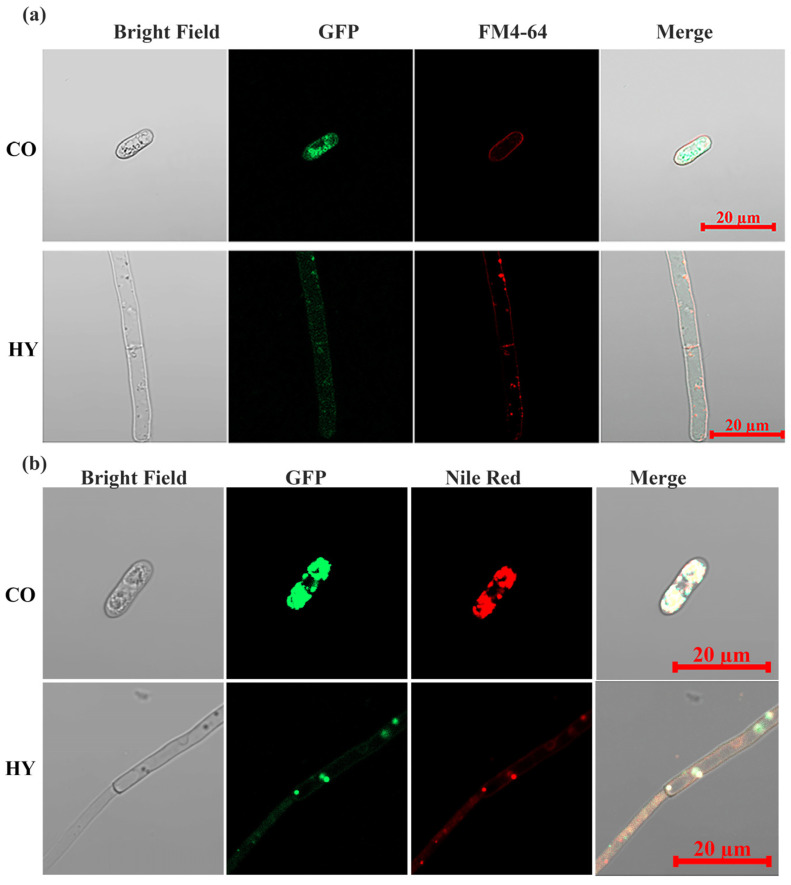
Subcellular localization of the CsPMA2 protein. (**a**) Observation of GFP signals and FM4-64 signals in the WT-CsPMA2-GFP strain. (**b**) Observation of GFP signals and Nile red signals in the WT-CsPMA2-GFP strain. All the photographs were taken under a confocal microscope (Zeiss, Oberkochen, Germany). HY, hyphae; CO, conidia.

To visualize the intracellular targeting of CsPMA2 in vivo, we generated a WT-CsPMA2-GFP strain expressing the CsPMA2-GFP fusion protein in *C. siamense* using the gpdA promoter, which was confirmed by PCR amplification, and GFP signals were captured under epifluorescence microscopy. Previous studies reported that the plasma membrane H^+^-ATPases PMA1 and PMA2 were localized to the plasma membrane in *F. graminearum* and *Ustilago maydis* [14,19]. Therefore, the WT-CsPMA2-GFP strain was stained with FM4-64 and then analyzed for GFP and FM4-64 signals using epifluorescence microscopy. The results showed that some GFP and FM4-64 signals clearly overlapped in mycelia and conidia (Figure 5a), which suggested that some CsPMA2 can localize to the plasma membrane. However, we also found strong green fluorescence signals on the intracellular LDs in conidia. Therefore, we treated the WT-CsPMA2-GFP strain with the lipid indicator Nile red. The results showed that green fluorescent signals overlapped with the red fluorescent signals of NR in the mycelia and conidia, including LDs and dispersed lipids (Figure 5b). These data indicated that CsPMA2 is localized to lipids both on the plasma membrane and intracellular membrane.

### 2.6. CsPMA2 Is Needed for Lipid Droplet Stability and the Content Balance of Some Lipid Species in C. siamense

**Figure 6:**
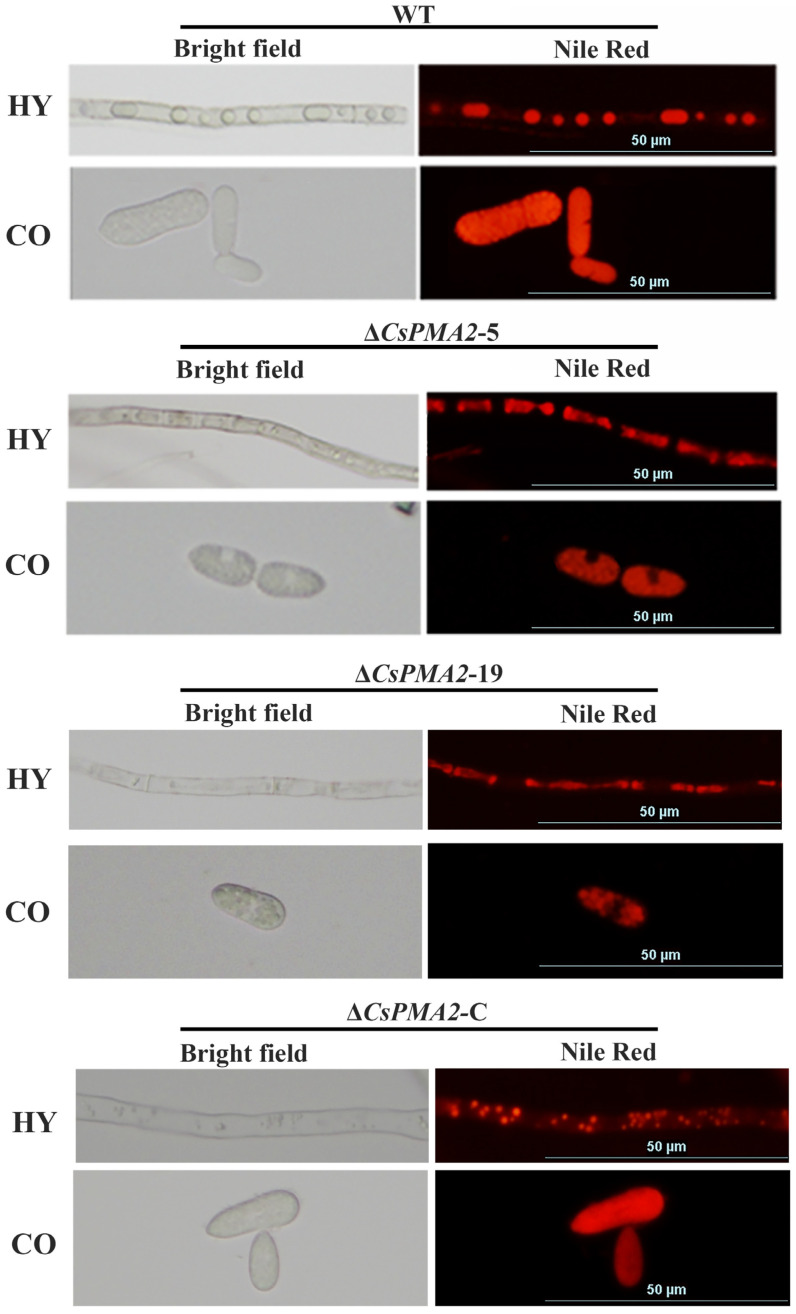
Comparison of lipid shape using Nile red staining among wild-type HN08, ∆*CsPMA2*-5, ∆*CsPMA2*-19, and Δ*CsPMA2*-C under a fluorescence microscope. HY, hyphae; CO, conidia.

Because CsPMA2 localizes to lipids, we investigated whether CsPMA2 is needed for lipid biogenesis. NR was used to label LDs in mycelia and conidia of the wild-type strain and mutants. The conidia are filled with numerous lipids, making it difficult to distinguish whether they are in the round-shaped LD phase or dispersed lipid phase in the wild type and mutants. However, a great number of round-shaped LDs were observed in mycelia of HN08, while dispersed lipids filled within the cytosol and far fewer round-shaped LDs were observed in the Δ*CsPMA2*-5 and Δ*CsPMA2*-19 mutants (Figure 6). The results suggested that LD stability was significantly influenced by *CsPMA2* gene deletion and that *CsPMA2* is needed for LD formation from the dispersed lipid phase in *C. siamense*.

**Figure 7:**
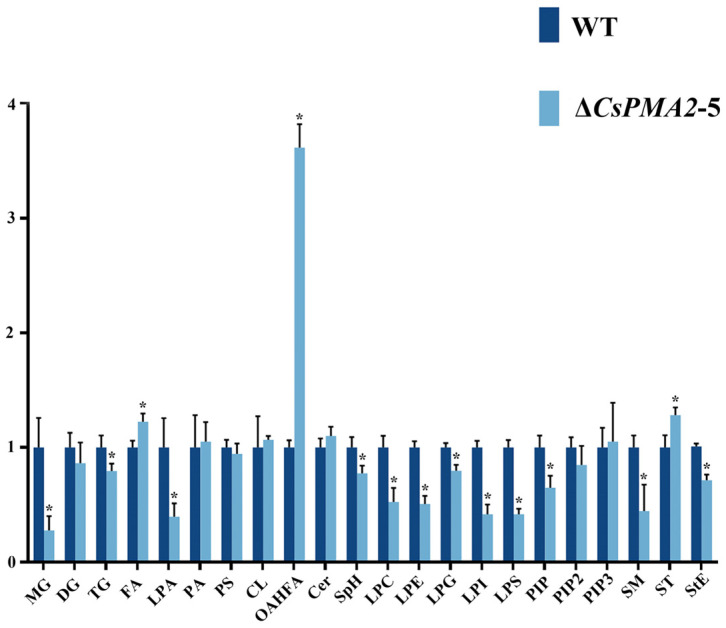
Relative content of lipid species in WT and Δ*CsPMA2* by lipidomic analysis. The content of lipids between HN08 and Δ*CsPMA2*-5 using liquid chromatography–mass spectrometry (LC–MS) technology. The relative content of lipid species was calculated as follows: define the abundance values of each lipid in the wild type as 1, and proportionally convert the abundance values of each lipid in the mutant to obtain the relative content of each lipid. Asterisks indicate statistically significant differences (*p* < 0.05). MG, Monoglyceride; DG, Diglyceride; TG, Triglyceride; FA, Fatty acid; LPA, Lyso-phosphatidic acid; PA, Phosphatidic acid; PS, Phosphatidylserine; CL, Cardiolipin; OAHFA, (O-acyl)-1-hydroxy fatty acid); Cer, Ceramides; SpH, Sphingoshine; LPC, Lyso-phosphatidylcholine; LPE, Lyso-phosphatidylethanolamine; LPG, Lyso-phosphatidylglycerol; LPI, Lyso-phosphatidylinositol; LPS, Lyso-phosphatidylserine; PIPs (PIP, PIP2, PIP3), phosphoinositides; SM, Sphingomyelin; ST, Sterol Lipids; StE, Stigmasteryl ester.

Lipid droplet stability is modulated by the presence of lipid or protein surfactants at the interface of the oil and water phases [45]. The major surfactants for LDs constitute a surface monolayer of amphiphilic molecules, such as phospholipids, in which the polar head groups are oriented toward the aqueous cytosol and the hydrophobic acyl chains are oriented toward the neutral lipid cores [45]. To evaluate whether CsPMA2 is involved in the phospholipid content in *C. siamense*, we compared the lipid content between HN08 and *ΔCsPMA2*-5 using liquid chromatography–mass spectrometry (LC–MS) technology (Figure 7 and Table S2). The results showed that 12 species of lipids in Δ*CsPMA2*-5 were significantly lower and 3 species of lipids were significantly higher than those in the wild-type strain. Among the lipid species of reducing content, monoglyceride (MG) and triglyceride (TG) are neutral lipids, and lysophosphatidylcholine (LPC), lysophosphatidylethanolamine (LPE), lysophosphatidylglycerol (LPG), lysophosphatidylinositol (LPI) and lysophosphatidylserine (LPS) are phospholipid species. These data demonstrated that CsPMA2 is needed for the synthesis of some species of lipids in *C. siamense.* We suggest that loss of *CsPMA2* may affect LD stability by reducing the surface amphiphilic molecule monolayer of LDs, such as phospholipids, to lower the LD surface tension.

### 2.7. Acid Stress Affects the Expression of CsPMA2 and the Accumulation of Lipid Droplets

**Figure 8:**
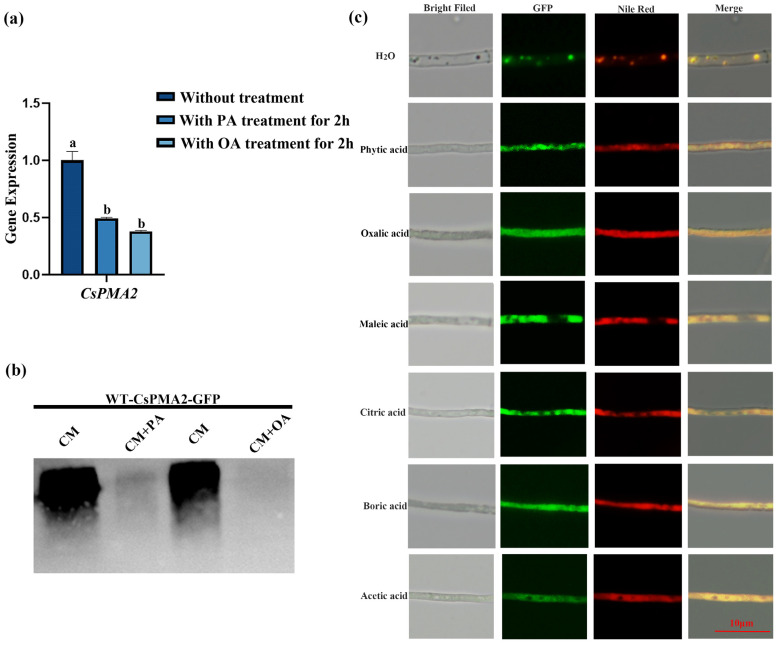
The expression of *CsPMA2*, the accumulation of lipid droplets and the localization of CsPMA2 in *C. siamense* with or without acid treatment. (**a**) The expression of *CsPMA2* mRNA in the wild-type strain with or without acid treatment was analyzed using qRT–PCR; (**b**) the content of CsPMA2 protein was analyzed in WT-CsPMA2-GFP with or without acid treatment using Western blotting. CM: Total protein was extracted from mycelia cultured on liquid CM medium; CM+PA: Total protein was extracted from mycelia with phytic acid treatment for 2 h; CM+OA: total protein was extracted from mycelia with Oxalic acid treatment for 2 h; (**c**) the lipid shape and the localization of CsPMA2 in the WT-CsPMA2-GFP strain with or without different acid treatments. Different letters indicate an extremely significant difference (*p* < 0.01) (one-way ANOVA and Duncan’s test).

To determine whether acid treatments affected the expression level of *CsPMA2*, qRT–PCR and Western blot assays were used. The results showed that the expression of *CsPMA2* mRNA with PA or OA treatments for 2 h was only 49.41% and 37.83% of that in the control group without treatment, respectively (Figure 8a). Western blot results also showed that the content of CsPMA2-GFP was significantly reduced in groups with PA or OA treatments (Figure 8b). These results indicated that acid stress can affect the expression of the *CsPMA2* gene in *C. siamense*.

Since CsPMA2 is involved in LD formation and the response to acid stress, is there a relationship between pH and LD formation? We treated the WT-CsPMA2-GFP strain with different acids, stained it with Nile Red dye, and then observed the GFP and RFP signals under a fluorescence microscope (Figure 8c). The results showed that the lipids were dispersed in cells and that droplet accumulation was severely inhibited with different acid treatments. The CsPMA2 protein was still localized on lipids, whether it was in the dispersed stage or in the LD stage. This finding indicated that acid stress can affect LD accumulation.

## 3. Discussion

Plasma membrane H^+^-ATPases play an important role in the pathogenicity of pathogenic fungi. Lipid droplets are also important for the infection of plant pathogenic fungi. However, the relationship between plasma membrane H^+^-ATPase, LD formation and virulence has not been reported. In this study, we cloned the plasma membrane H^+^-ATPase coding gene *CsPMA2* from *C. siamense* and determined that *CsPMA2* was dispensable for the nutritional growth of *C. siamense* mycelia but essential for conidial size, conidial germination, appressorial development and virulence of *C. siamense*. Interestingly, we demonstrated that CsPMA2 localized to lipids on the plasma membrane and intracellular membrane and was involved in LD formation and the response to acid stress. Overall, our data revealed that the plasma membrane H^+^-ATPase CsPMA2 is involved in the regulation of LD formation and affects appressorial development and virulence in *C. siamense*. These findings demonstrated the relationship between plasma membrane H^+^-ATPase, LD formation and virulence and will help us further understand the mechanism of plasma membrane H^+^-ATPases in virulence in *C. siamense*.

It was reported that plasma membrane P-type ATPase is a type of transport protein localized in the plasma membrane and is responsible for pumping hydrogen proteins from the cytoplasm to the extracellular space to maintain intracellular pH and ion homeostasis [7,8,9]. There are two plasma membrane H^+^-ATPases, PMA1 and PMA2, in fungi [10,11,12]. Many studies have shown that PMA1 is localized in lipid microdomains of the plasma membrane and plays an important role in cell growth and virulence in pathogenic fungi [16,17]. However, *PMA2* is generally believed to have little effect on the growth and pathogenicity of fungi due to its extremely low expression in cells [22]. For instance, it has been reported that *FgPMA2* in *F. graminarium* and *LmPMA2* in *L. maculans* do not affect its vegetative growth, sexual and asexual development, or virulence [19,21]. In this study, we obtained *CsPMA2* gene deletion mutants but not *CsPMA1* after several attempts. We hypothesized that *CsPMA1* is important for mycelial growth or is lethal for *C. siamense*. Furthermore, we verified that *CsPMA2* was dispensable for the nutritional growth of *C. siamense* mycelia but essential for conidial size, conidial germination, appressorial development and virulence of *C. siamense* in this study, which was consistent with the observation of *ChPMA2* in *C. higginsiamum* [23]. These results suggest that PMA2 functions vary in different pathogenic fungi, especially in pathogenicity.

The function of PMA2, in regard to virulence, varies in different pathogenic fungi, which may be due to the different pathogenic mechanisms in different fungi. In the *Colletotrichum* genus, the appressorium is a specialized infection structure that penetrates the cuticles of its host. Lipid droplets are a universal reservoir of neutral lipids and play a crucial role in the infection process of plant pathogens mediated by appressoria [29,46,47,48]. Lipid biosynthesis produces glycerol, which is important in fueling turgor pressure necessary for the germination and penetration of plant hosts by fungi [30]. In this study, we found that deletion of *CsPMA2* reduced the appressorial turgor pressure and enlarged the pore size of the cell wall of mutant appressoria. We also found that CsPMA2 is not only localized in the plasma membrane, as reported previously, but is also widely distributed in intracellular LDs. Deletion of *CsPMA2* resulted in significant inhibition of LD accumulation and loss of the balance of some lipid species in *C. siamense*. These data imply that CsPMA2 may affect fungal virulence by balancing the lipid content and LD stability to maintain the ability of the appressoria to penetrate the host cuticle.

Lipid droplets are a universal reservoir of neutral lipids for energy or membrane synthesis. Previous studies have shown that there may be an interplay between lipids and PMA1 in fungi. PMA1 is reported to localize in lipid microdomains of the plasma membrane, and studies in *Saccharomyces cerevisiae* have shown that the efficient function of PMA1 is greatly dependent on sphingolipids [36,37]. However, the relationship between plasma membrane H^+^-ATPase and LD formation remains unclear. In this study, we demonstrated that CsPMA2 was important for LD stability. How does CsPMA2 affect the stability of LDs? Lipid droplet stability is modulated by the presence of lipid or protein surfactants at the interface of the oil and water phases. Surfactants lower the LD surface tension. We considered that CsPMA2 did not act as a protein surfactant, like Cap20, at the interface of the oil and water phases [24,34], because CsPMA2 proteins not only localized to LDs but also to dispersed lipids, as we observed. Therefore, we speculated that the loss of *CsPMA2* may affect LD stability by affecting the presence of lipid surfactants. It was reported that the major surfactants for LDs constitute a surface monolayer of amphiphilic molecules, such as phospholipids, in which the polar head groups are oriented toward the aqueous cytosol and the hydrophobic acyl chains are oriented toward the neutral lipid cores [45]. Previous studies have shown that cytosolic pH, coregulated by plasma membrane H^+^ ATPase Pma1 and vacuolar ATPase (V-ATPase) [27], affects the electrostatic interaction and affinity of Opi1 (a major transcription factor repressor) toward PA in the ER. And the alteration in this interaction will impact the induction effect of Opi1 on INO1 [49] and the subcellular localization of Opi1, leading to its inhibition of the Ino 2/4 transcriptional activator complex within the nucleus. Consequently, this induces the expression of phospholipid metabolism genes such as CDS1 [50], thereby influencing the pathway of phospholipid biosynthesis. Furthermore, Opi1′s leucine zipper domain also plays a regulatory role in the lipid synthesis pathway [51]. According to our results of the content of various lipids between wild type and Δ*CsPMA2*, deletion of the *CsPMA2* gene resulted in a significant reduction in the content of several phospholipid species (including LPC, LPE, LPG, LPI and LPS), sphingolipids (SM and SPH) and neutral lipids (MG and TG). This finding is consistent with our speculation that loss of *CsPMA2* may affect LD stability by reducing the content of surface amphiphilic molecule monolayers of LDs, such as phospholipids, to lower the LD surface tension.

In conclusion, this study cloned and functionally analyzed the plasma membrane transport H^+^ ATPase CsPMA2 in *C. siamense*. CsPMA2 is localized to lipids on the plasma membrane and intracellular membrane, which is important for balancing the content of some lipid species and LD stability. CsPMA2 is also involved in conidial germination, appressorial development and virulence in *C. siamense*. Our study not only enriches the understanding of the functions of the plasma membrane H^+^-ATPase PMA2 but also helps to further reveal the regulatory mechanism underlying LD formation in *C. siamense*.

## 4. Materials and Methods

### 4.1. Strains and Culture Conditions

The wild-type *C. siamense* strain HN08 was isolated from diseased leaves of rubber trees in a rubber tree farm in Hainan province and was identified as *C.siamense*, which was described previously [52]. The HN08 strain and the corresponding mutants were cultured on potato dextrose agar (PDA) plates at 25 °C and stored in sterile water at room temperature. The mutant strains Δ*CsPMA2-*5 and Δ*CsPMA2-*19, the complementary strain Δ*CsPMA2-*C and the CsPMA2-GFP fusion-expressing strain WT-CsPMA2-GFP were derived from HN08. The strains were grown in liquid potato dextrose medium (PD; 20 g/L potato, 20 g/L glucose), and the conidia were collected for phenotypic tests such as hyphal growth rate and stress sensitivity. For extraction of DNA and RNA, mycelia were cultured in liquid complete medium (CM; 0.6% yeast extract, 0.1% casein acid hydrolysate, and 1% sucrose) for 3–5 d in the dark at 28 °C.

### 4.2. CsPMA2 Gene Cloning and Sequence Analysis

We used the sequences of the plasma membrane transport H^+^ P-type ATPases ChPMA1 (Accession KP261085) and ChPMA2 (Accession KP180423) reported in *C. higginsiamum* to local BLAST search in the entire genome database of *C. siamense* HN08 and obtained the sequences of the plasma membrane transport H^+^ P-type ATPases CsPMA1 and CsPMA2 of *C. siamense*. The primers CsPMA2-F and CsPMA2-R (all primers used in this study are listed in Table S1) were designed for amplification of the whole coding sequences of *CsPMA2* DNA and cDNA. The cDNA sequence was transformed into an amino acid sequence and analyzed using the Modular Architecture Research Tool (SMART, http://smart.embl-heidelberg.de/, accessed on 15 May 2022). The phylogenetic tree was constructed by the maximum likelihood method using MEGA 11.0.10 with 1000 bootstrap values.

### 4.3. Targeted Disruption of CsPMA2, Complementation and Protein Subcellular Localization

Homologous recombination was performed for *CsPMA2* gene deletion. The sequences of primers used are provided in Supplementary Table S1. Primer pairs were designed as shown in the schematic diagram in Figure S1a. First, the 5′ and 3′-flanking regions (approximately 0.7 kb) of *CsPMA2* were amplified using primers CsPMA2-UF/CsPMA2-UR and CsPMA2-DF/CsPMA2-DR from genomic DNA. CsPMA2-UR and CsPMA2-DF contained the linker sequences of the chlorimuron resistance gene (*ILV1*). Second, whole *ILV1* was amplified by PCR using the primer pair ILV1-F/ILV1-R and the pCX62-S plasmid as a template. Finally, the first- and second-round PCR products were used as templates, and enriched fusion fragments were obtained by fusion PCR amplification using CsPMA2-UF and CsPMA2-DR as primers. The PCR products were then transformed into protoplasts of the wild-type strain HN08 as reported [52].

For the construction of complementation and GFP fusion-expressing strains, cDNA sequences including the full open reading frame of the *CsPMA2* gene without a terminal code were amplified using the primer pair CsPMA2-GFP-F/CsPMA2-GFP-R and cloned and inserted into the pBAR-GFP plasmid (containing the gpdA promoter and the hygromycin transferase gene *HPH*) using the ClonExpress II one step cloning kit C115 (Vazyme Biotech, Nanjing, China). Then, the recombinant plasmid pBAR-CsPMA2-GFP was transformed into protoplasts of the Δ*CsPMA2*-5 mutant and wild-type HN08 to obtain the complementary strain Δ*CsPMA2-*C and subcellular localization strains WT-CsPMA2-GFP. Transformants were selected on PDS medium (PDA medium with 1 M sucrose added) supplemented with 600 µg/mL hygromycin.

### 4.4. Phenotypic Characterization

To measure conidial germination, appressorial formation, and appressorium turgor pressure and cell wall porosity, 20 μL of spore suspensions (1 × 10^5^ conidia/mL) was prepared and dropped onto hydrophobic slides and incubated at 28 °C according to Song et al. [52]. Conidial germination rates were observed at 0, 2, 4, and 6 h, and the rates of appressorial formation and malformation were analyzed at 16 h. Appressorial turgor pressure and cell wall porosity were estimated by counting the number of appressorium undergoing cytorrhysis and the ratio of plasmolysis treated with 0.8 g/mL PEG8000 as previously described [34,53]. Three independent experiments were performed.

To assay the effects of different stress conditions on the tested strains, 10 µL of spore suspensions (1 × 10^5^ spores/mL) was inoculated on CM plates supplemented with sorbitol (1 M) and NaCl (0.5 M), Congo Red (CR, 400 µg/mL), PA (0.1% phytic acid) and oxalic acid (16 mM). Colony diameters were measured and photographed after incubation for 3 days at 28 °C. CM plates were used as controls. All experiments were carried out in triplicate.

To test virulence, 20 µL of spore suspensions (1 × 10^5^ spores/mL) of the tested strains were dropped on detached tender leaves with or without wounding as described by Song et al. [52]. Each treatment was repeated 3 times, and 30 leaves were inoculated per treatment. The disease lesions were measured and photographed 5 days after inoculation.

### 4.5. Fluorescence Microscopy Observation

To determine the localization of CsPMA2, the plasmid pBAR-CsPMA2-GFP was transformed into wild-type HN08, and the subcellular localization strain WT-CsPMA2-GFP was obtained. Then, the mycelia, conidia and appressoria of transformants were observed by using a Zeiss confocal microscope (Zeiss Oberkochen Germany). The fluorescence channel of GFP was used for observation.

To determine the localization of lipids, mycelia and conidia of strains were stained with Nile Red solution (Solarbio, Beijing, China) as described by Wang et al. [35]. The fluorescence channel of RFP was used for observation. For FM4-64 staining, 100 µL of FM4-64 staining solution (concentration 5 µg/mL Sigma–Aldrich, St. Louis, MO, USA) was added dropwise to the slides and stained for 10 min at room temperature under dark conditions. The fluorescence channel of RFP was used for observation.

WT-CsPMA2-GFP transformants were incubated for 2 h in aqueous solutions with different acids (0.1% phytic acid; 16 mM oxalic acid; 16 mM maleic acid; 16 mM citric acid; 16 mM boric acid; and 16 mM acetic acid). Sterile water was used as a control. Then, mycelium was washed twice with sterile water, stained with Nile red dye, and finally washed twice with sterile water before observing the formation of LDs under a fluorescence microscope.

### 4.6. Lipid Content Determination

The measurement of lipid content was entrusted to Shanghai Personalbio Technology Co., Ltd. (Shanghai, China). The lipid content of the WT HN08 and ∆*CsPMA2-5* mutant strains was quantified by liquid chromatography–mass spectrometry (LC–MS). Reversed-phase chromatography was selected for LC separation using a CSH C18 column (1.7 µm, 2.1 mm× 100 mm, Waters). The lipid extracts were redissolved in 200 µL 90% isopropanol/acetonitrile and centrifuged at 14,000× *g* for 15 min. Finally, 3 µL of sample was injected. Solvent A was acetonitrile–water (6:4, *v*/*v*) with 0.1% formic acid and 0.1 mM ammonium formate, and solvent B was acetonitrile–isopropanol (1:9, *v*/*v*) with 0.1% formic acid and 0.1 mM ammonium formate. The initial mobile phase was 30% solvent B at a flow rate of 300 μL/min. It was held for 2 min and then linearly increased to 100% solvent B in 23 min, followed by equilibrating at 5% solvent B for 10 min.

Mass spectra were acquired by Q Exactive Plus in positive and negative mode. The ESI parameters were optimized and preset for all measurements as follows: source temperature, 300 °C; capillary temperature, 350 °C; ion spray voltage, 3000 V; S-Lens RF level, 50%; and scan range of the instruments, *m*/*z* 200–1800.

### 4.7. Quantitative Real-Time PCR (qRT–PCR) Analysis and Western Blot Analysis

Wild-type strain HN08 was incubated in complete medium (CM; 0.6% yeast extract, 0.1% casein acid hydrolysate, and 1% sucrose) at 28 °C for 3 days. The treatment group was treated with 0.1% phytic acid and 16 mM oxalic acid for 2 h before RNA extraction, while the control group was treated without any treatment. Then, the mycelia were collected, and total RNA of the mycelia of the HN08 strains was extracted using the RNAprep Pure Plant Kit (Tiangen, Beijing, China). cDNA synthesis was performed with TransScript One-Step gDNA Removal and cDNA Synthesis SuperMix (TransGen Biotech, Beijing, China). The expression levels of the *CsPMA2* gene before and after treatment with phytic acid and oxalic acid were quantified by quantitative real-time PCR (qRT–PCR) performed with an ABI7500 sequence detection system (Applied Biosystems, Waltham, MA, USA). Reactions were performed in a total volume of 10 µL using the SYBR Premix Dimer Eraser Kit (Takara, Beijing, China). All of the reactions were repeated in at least three independent pools in three sets of biological replicates.

Strain WT-CsPMA2-GFP containing the CsPMA2-GFP fusion protein was incubated in complete medium (CM; 0.6% yeast extract, 0.1% casein acid hydrolysate, and 1% sucrose) at 28 °C for 3 days. The treatment group was treated with 0.1% phytic acid and 16 mM oxalic acid for 2 h before protein extraction. Then, mycelia with or without treatments were harvested and washed with phosphate-buffered saline (PBS) for protein extraction. Approximately 100 mg of mycelia was used for protein extraction. After shaking at 4 °C for 30 min, the mixture was centrifuged at 12,000–14,000× *g* in a low-temperature centrifuge for 10 min at 4 °C. Fifteen microliters of each sample was loaded onto 10% SDS–PAGE gels. Proteins separated on gels were transferred to polyvinylidene fluoride (PVDF) membranes. Monoclonal anti-GFP was used at a 1:1000 dilution for immunoblot analyses. Chemiluminescence detection after incubation with a secondary antibody.

## Data Availability

The data that support the findings of this study are available from the corresponding author upon reasonable request.

## Supplementary Materials

The following supporting information can be downloaded at: https://www.mdpi.com/article/10.3390/ijms242417337/s1.
