# Supplementary material for: The Plasma Membrane H+ ATPase CsPMA2 Regulates Lipid Droplet Formation, Appressorial Development and Virulence in Colletotrichum siamense
Source: Int J Mol Sci. 2023 Dec 11;24(24):17337. doi: 10.3390/ijms242417337 (PMC10743824; doi:10.3390/ijms242417337)
Supplement: Supplementary file 1 [file ijms-24-17337-s001.zip › ijms-2739495-supplementary.pdf]

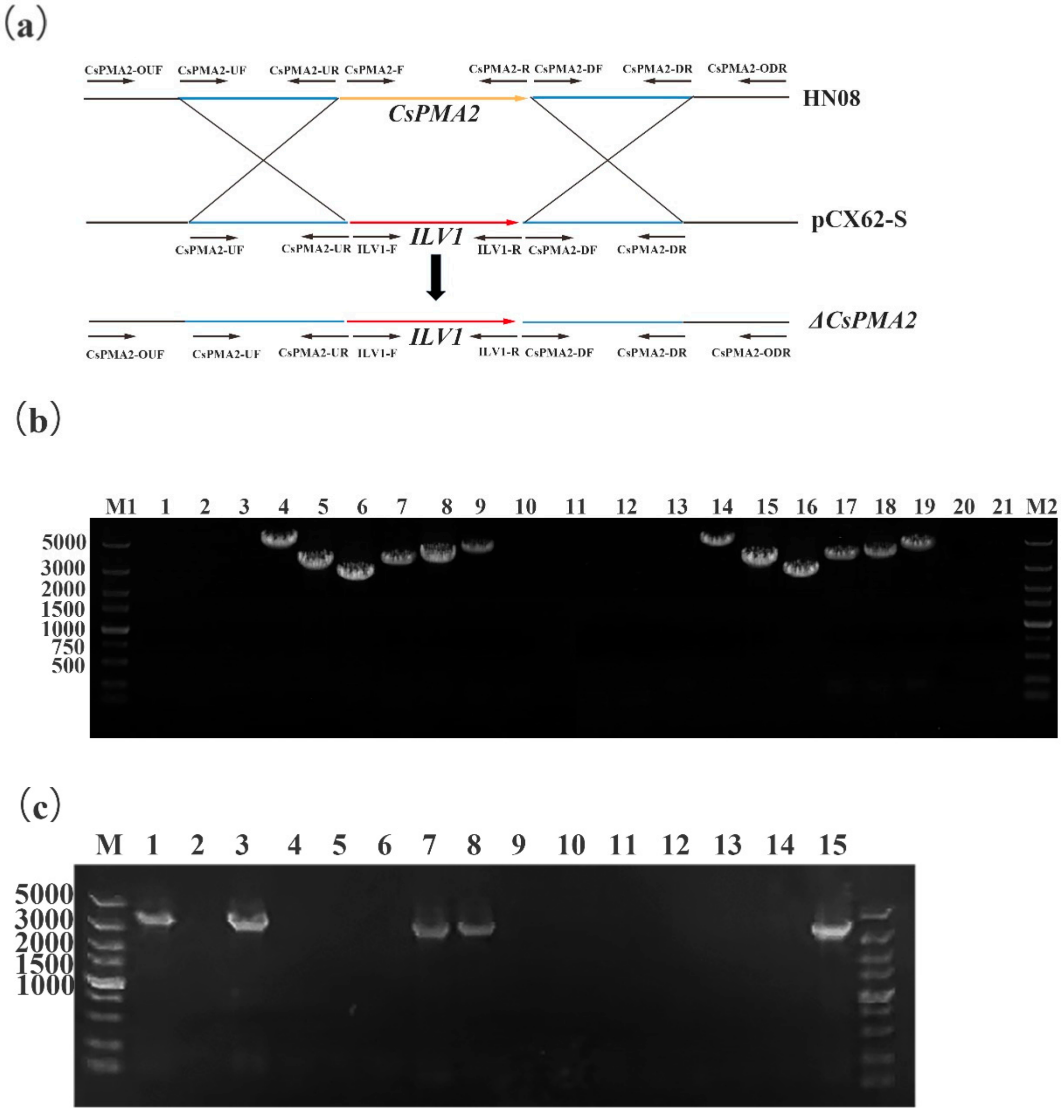

Figure S1: Schematic diagram of CsPAM2 gene knockout and verification of the gene deletion mutants ∆*CsPMA2*-5 and ∆*CsPMA2*-19 and the complemented transformant ∆*CsPMA2*-C. **(a)** Schematic diagram of the construction of the *CsPMA2* gene deletion mutant and primers. **(b)** PCR verification of the *CsPMA2* gene deletion mutants. The internal sequence of the *CsPMA2* gene could be amplified by the primers CsPAM2-F/CsPMA2-R in the wild-type (Lanes 5 and 15) and but not in the Δ*CsPMA2*-5 and *ΔCsPMA2*-19 strains (Lanes 10 and 20). The sequence of *the ILV1* gene could be amplified by primers ILV1-F/ILV1-R in the ∆*CsPMA2*-5 and ∆*CsPMA2*-19 strains (Lanes 6 and 16) but not in the wild type (Lanes 1 and 11). The sequence with the upstream sequence of *CsPMA2* and partial *ILV1* gene sequence amplified by CsPMA2-OuF/ILV1-R and the fragment with the downstream sequence of *CsPMA2* and partial *ILV1* gene sequence amplified by ILV1- F/CsPMA2-ODR are shown in ∆*CsPMA2* (Lanes 7,8 and 17,18) and not the wild type (Lanes 2,3 and 12,13). The PCR products amplified from mutant ∆*CsPMA2*-5 and ∆*CsPMA2*-19 and wild-type HN08 using primers CsPMA2-OU-F/CsPMA2-OD-R were sequenced, yielding a 4801 bp fragment from ∆*CsPMA2*-5 and ∆*CsPMA2*-19 (Lanes 9 and 19) and a 5338 bp fragment from HN08 (Lanes 4 and 14). Lanes M1 and M2 are the DNA DL5000 marker. **(c)** PCR verification of the *CsPMA2* gene complemented strain. The internal sequence of the *CsPMA2* gene could be amplified by the primers CsPAM2-F/CsPMA2-R in the wild-type (Lane 1) and ∆*CsPMA2*-C strains (Lanes 3, 7, 8 and 15).

Table S1: List of primers used in this study

Primer name	Sequence(5'-3')
CsPMA2-OUF	CATCGTCAAGTCACTTCAGAGTTCT
CsPMA2-ODR	CTTGCTCTTCCCACGAAATATCGT
CsPMA2-UF	CTATACGTACTGCTTGCTGC
CsPMA2-UR	AGATGTGGGGCACTGTGGCGTTGGCACGGTGACGAGGGATTACAGTC
CsPMA2-DF	TATTGCACGGGAATTGCATGCTCTCACACCGCATTGATCTCGCAAAT
CsPMA2-DR	AGCCGGAGTTGCGAATCATG
CsPMA2-F	ATGGCTCCCTTCAAAATGGC
CsPMA2-R	TTAACCCTCCTCGACAACGGT
ILV1-F	GTGCCAACGCCACAGTGCCCCACA
ILV1-R	GTGAGAGCATGCAATTCCCGTGCAATA
CsPMA2-GFP-F	GACCTCGACTCTAGAGGATCCATGGCTCCCTTCAAAATGGC
CsPMA2-GFP-R	GCCCTTGCTCACCATGGATCCACCCTCCTCGACAACGGTG
GFP-F	GACCTCGACTCTAGAGGATCCATGGTGAGCAAGGG
GFP-R	GATAAGCTTGATATCGAATTCTTACTTGTACAGCTCGTCCATGCC
CsPMA2qRT-F	AATACAAGTCTCTCGTCCGCTAC
CsPMA2qRT-R	TTCTTCCAGAACATCCATGAGGG
Actin-F	GATTTGGCACCACACCTTCTACA
Actin-R	TCTCTGTTGGACTTGGGGTTGAT

Table S2: The abundance values of 41 species of lipids were analyzed by LC–MS in the wild-type strain HN08 and Δ*CsPMA2*.

Lipid Name	Abbreviation	Mean abundance value of HN08	Mean abundance value of Δ*CsPMA2*
Ceramides	Cer	1346511812	1158766294
N-acetylhexosyl Ceramide	CerG2GNAc1	556632.5197a*	374701.0073b
Dihexosyl N-acetylhexosyl Ceramide	CerG3GNAc1	226462.7647a	377673.9201b
Ceramides phosphate	CerP	31621582.83a	22643771.17b
Cholesterol Ester	ChE	325502.2752a	214878.5003b
Cardiolipin	CL	67819928.83	72297809.49
Coenzyme Q	Co	4561516.783a	5585603.391b
Diglyceride	DG	4934102771	4260495197
Fatty acid	FA	2912330.73a	3233884.484b
Disialo dihexosyl ceramide	GD2	564509.3981	637640.6213
Monosialo tetrahexosyl ceramide	GM1	160585.1144a	212600.4301b
Monosialo trihexosyl ceramide	GM3	1990515.479a	1496834.54b
Trisialo trihexosyl	GT3	109145.8078a	9591.808298b
Hexosyl ceramide	Hex1Cer	614265533.6a	502980530.1b
Hexosyl ceramide	Hex2Cer	24890650.61	26251228.47
Hexosyl ceramide	Hex3Cer	1215496.125a	972695.2064b
Lysophosphatidic acid	LPA	89242110.49a	35235779.36b
Lysophosphatidylcholine	LPC	63635906.72a	39815180.21b
Lysophosphatidylethanolamine	LPE	51261398.75a	26021878.22b
Lysophosphatidylglycerol	LPG	3013221.5a	2404156.894b
Lysophosphatidylinositol	LPI	5732765.047a	2389841.554b
Lysophosphatidylserine	LPS	1732980.732a	722350.8269b
Monoglyceride	MG	19015855.94a	5300616.762b
(O-acyl)-1-hydroxy fatty acid	OAHFA	1005072.105a	3633645.796b
Phosphatidic acid	PA	709285624.1	745348841.2
Phosphatidylcholine	PC	463320591.5	430672970.4
Phosphatidylethanolamine	PE	970525790.2	845063093.8
Phosphatidylglycerol	PG	523526693.6	522569625.1
Phytosphingosine	phSM	1495092.85a	696925.7719b
Phosphatidylinositol	PI	151998785.4	174718413.1
Phosphatidylinositol(4)phosphate	PIP	33079959.75a	21457423.18b
Phosphatidylinositol(4,5)bisphosphate	PIP2	14764771.55	12536038.19
Phosphatidylinositol(3,4,5)triphosphate	PIP3	3770608.987	3964929.506
Phosphatidylserine	PS	29722781.09	28003258.27
Sphingomyelin	SM	4191805.008a	1868527.479b

Sphingosine	SPH	47814328.03a	37077293.29b
Sulfatide	ST	28677252.24a	37718920.26b
Stigmasterol ester	StE	4545068.265a	3707215.74b
Triglyceride	TG	24333823667a	20155468666b
Wax exters	WE	205999710.6	202132482.6
Zymosterol	ZyE	5335758.669	5197885.609

*Different letters indicate an extremely significant difference (p<0.01) (One-way ANOVA and Duncan's test).
